# Mechanical effects of canes on standing posture: beyond perceptual information

**DOI:** 10.1186/s12984-022-01067-7

**Published:** 2022-09-10

**Authors:** Marta Russo, Jongwoo Lee, Neville Hogan, Dagmar Sternad

**Affiliations:** 1 Department of Biology, Northeastern University, Boston, MA USA; 2 Department of Neurology, Tor Vergata Polyclinc, Rome, Italy; 3 Department of Mechanical Engineering, Massachusetts Institute of Technology, Cambridge, MA USA; 4 Department of Brain and Cognitive Sciences, Massachusetts Institute of Technology, Cambridge, MA USA; 5 Department of Electrical and Computer Engineering, Northeastern University, Boston, MA USA; 6 Department of Physics, Northeastern University, Boston, MA USA

**Keywords:** Postural control, Standing balance, Haptic information, Mechanical support, Stability

## Abstract

**Background:**

Numerous studies showed that postural balance improves through light touch on a stable surface highlighting the importance of haptic information, seemingly downplaying the mechanical contributions of the support. The present study examined the mechanical effects of canes for assisting balance in healthy individuals challenged by standing on a beam.

**Methods:**

Sixteen participants supported themselves with two canes, one in each hand, and applied minimal, preferred, or maximum force onto the canes. They positioned the canes in the frontal plane or in a tripod configuration. Statistical analysis used a linear mixed model to evaluate the effects on the center of pressure and the center of mass.

**Results:**

The canes significantly reduced the variability of the center of pressure and the center of mass to the same level as when standing on the ground. Increasing the exerted force beyond the preferred level yielded no further benefits, although in the preferred force condition, participants exploited the altered mechanics by resting their arms on the canes. The tripod configuration allowed for larger variability of the center of pressure in the task-irrelevant anterior–posterior dimension. High forces had a destabilizing effect on the canes: the displacement of the hand on the cane handle increased with the force.

**Conclusions:**

Given this static instability, these results show that using canes can provide not only mechanical benefits but also challenges. From a control perspective, effort can be reduced by resting the arms on the canes and by channeling noise in the task-irrelevant dimensions. However, larger forces exerted onto the canes can also have destabilizing effects and the instability of the canes needs to be counteracted, possibly by arm and shoulder stiffness. Insights into the variety of mechanical effects is important for the design of canes and the instructions of how to use them.

## Background

Over the past decades many neuroscientists focused their attention on the role of sensory information in the control of standing posture. Not surprisingly, visual input together with vestibular and proprioceptive information significantly contribute to maintaining balance [1, 2]. Less straightforward is the role of haptic information, obtained through touching a surface or holding the hand of another person. A seminal study showed that light touch of the fingertip on an earth-fixed surface significantly reduced the motion of the center of pressure (CoP) during standing [3]. Increasing the amount of force applied on that surface had only minimal effect on the CoP motion, although the forces tested remained very small. Further, most subsequent studies on light touch continued to test participants’ ability to balance using earth-fixed supports that were stationary, both with unilateral support [4] and with bilateral support [5]. The situation is different when relying on a supporting device that itself may be moving. For example, holding a cane or the hand of another person does not present a stable stationary support. Yet, as frequently observed, seeking support from such nonstationary devices or another person do appear to provide stability [6].

A small number of studies have attempted to understand the assistance provided by unstable or unreliable support during standing or also walking. Blind individuals, who frequently use a ‘white cane’, report that they gain useful stabilization through this haptic information. Jeka and collaborators confirmed that touch with a cane, i.e., indirect contact of a surface through a hand-held stick, reduced postural sway [7]. In the same vein, a recent study examined bimanual support gathered via two ropes held in each hand and anchored to the ground [8]. The researchers compared CoP motion in this scenario with bimanual light touch of two earth-fixed supports. The latter proved superior in reducing CoP variability, albeit both support conditions were superior to standing with the hands free. In addition, it has been shown that light touch even of a curtain, a highly unreliable reference, benefitted postural stability [9, 10]. A subsequent study by Bryanton and colleagues confirmed that such unreliable touch reference attenuated CoP motion, although they also reported an increase in CoP variability when the reference was perturbed [11]. They speculated that an unrealible touch reference might engage a reweighting mechanism of tactile perceptual cues.

The role of light touch becomes even more important when standing on an unstable surface such as a foam pad or a beam elevated above ground [12, 13]. When standing on a light foam pad, any displacement of the touch support required balance corrections through enhanced activity of the tibialis anterior [13]. When standing on an elevated beam, earth-fixed light touch reduced CoP motion both with and without visual input [12]. The same study also showed that bimanual touch improved postural balance, especially when standing on a higher beam where fear of falling became an issue. This wide range of studies confirmed the role of perceptual information through light touch.

While these results are convincing, little attention has been paid to mechanical aspects that are undoubtedly also present. The force levels that have been investigated in these previous studies of light touch typically ranged from < 1 N to about 10 N. At such low force levels, mechanical benefits are indeed likely to be subordinate. However, support from a surface or cane also affords leaning on it and becomes relevant when standing on a challenging support surface, such as on a narrow beam. Hence, the aim of the present study was to investigate the mechanical contribution to standing balance by support through hand-held canes. Note that perceptual and mechanical influences cannot be strictly separated as neither of the two can be eliminated. However, we follow the traditional experimental approach of enhancing one aspect over the other and then comparing performance to baseline conditions.

To enhance the need for mechanical support, this study challenged postural stability by asking participants to stand on a narrow beam on the ground. With one foot placed behind and in line with the other, the base of support was strictly limited to the beam dimensions, in particular to its width. Then, to help participants to maintain balance, they held two canes, one in each hand, and placed them on the ground. First, we quantified to what extent the beam indeed affected postural stability, comparing the variability of the CoP and the center of mass (CoM) between standing on the ground and on the beam. We expected that the beam induced significant instability. We then assessed the contribution of cane support when standing on the beam compared to without canes. We expected that when standing posture is assisted via canes, the variability of the CoM and the CoP is significantly reduced (Hypothesis 0).

To enhance the mechanical contributions to postural stability compared to the perceptual contributions shown from cane support in earlier studies, the present study increased and manipulated the forces exerted on the cane to examine their effect on postural stability. We reasoned that greater forces applied on the canes would have significant mechanical effects on postural stability; specifically, we expected that the variability of the CoP and also the CoM would be reduced (Hypothesis 1).

From a mechanical perspective the configuration of the canes can have a significant effect on postural stability. Thus, participants were instructed to place the canes in two different arrangements. As standing on a beam in tandem stance has a large destabilizing effect in the medio-lateral direction, participants can directly control and compensate for their angular momentum in the frontal plane [14]. Therefore, in a first configuration, participants were instructed to hold the canes symmetrically with the arms outstretched to the sides, i.e., in the frontal plane. In a second configuration, the two canes were placed diagonally in front of the body. While it will contribute to the medio-lateral direction, it will also allow more room for motion in the antero-posterior direction. Due to the triangular base of support, we reasoned that this ‘tripod’ condition would significantly improve postural balance (Hypothesis 2).

Nevertheless, canes present an additional challenge: a vertical cane on the ground is an inverted pendulum that is inherently unstable. When applying higher forces axially onto the canes, any small deviations from this axial force direction can deflect the cane, leading to it falling over. As physiological noise is known to increase proportionally with isometric force, higher forces exerted on the canes can have the opposite effect and destabilize posture [15]. It was previously shown that exerting a compression force on a mechanical rod induced mechanical instability, such that the cane could be easily pushed over with any small perturbation [16, 17]. Based on these effects, we expected that exerting higher forces onto the canes would induce more variability (Hypothesis 3).

To evaluate these intricate mechanical effects, ground reaction forces were measured both at the feet and at the canes. This allowed separate quantification of the center of pressure at the feet and over the total support area spanned by the feet and canes. With additional 3D kinematic recordings, we also assessed the motion of the CoM and of the hands on the canes.

## Methods

### Participants

Sixteen participants (7 females, 9 males, between 19 and 36 years, BMI: 24.58 ± 5.04 kg/m^2^) with no history of neurological conditions and normal or corrected-to-normal vision took part in the experiments upon signing the informed consent form. The study was approved by the Institutional Review Board of Northeastern University in accordance with the Declaration of Helsinki (IRB# 18-01-19).

### Experimental apparatus

**Fig. 1:**
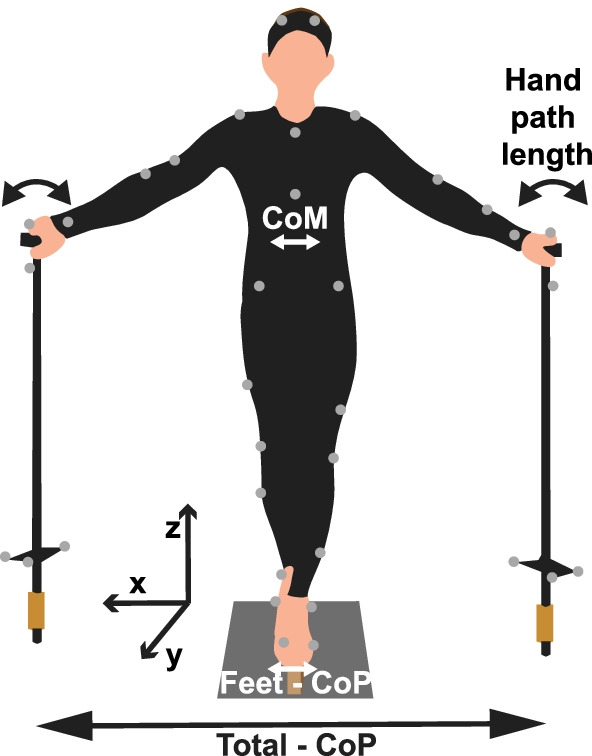
Experimental setup. Participants stood on a beam that was placed in a fixed position on a force plate, holding a cane in each hand. A set of 43 light-reflective markers measured displacements of the full body and the canes in 3D. The canes were instrumented with two 6D force/torque sensors at the bottom of each cane. The sketch shows the planar cane configuration where the two canes were placed to be on one line with the feet (planar configuration). In the tripod configuration, the canes were placed 45 degrees to the front to form a triangle with the feet

Participants stood on a narrow wooden beam (width 3.65 cm, height 7.62 cm) that was placed on the floor on top of a force plate (AMTI, Watertown, MA, USA, Fig. 1). They held two aluminum canes, one in each hand, to support themselves (length 117 cm, mass 680 g). The two canes were instrumented with a 6-DOF load cell at the bottom of each cane to measure the forces applied to the canes (MCW-500 Walker Sensors, AMTI Watertown, MA, USA). All force data were recorded at 500 Hz sampling rate. To record the participants’ movements in 3D, whole-body kinematics was recorded by 12 optical cameras at a sampling rate of 100 Hz (Qualisys, Göteborg, Sweden). Each participant was equipped with a standard biomechanical set of 43 reflective markers, following the C-Motion Plug-In Gait marker set. To track the orientation of the canes in 3D, 4 additional markers were attached to each cane.

### Experimental protocol

Participants were asked to stand barefoot in tandem stance on the narrow beam and maintain their gaze fixed to a point marked on the wall. They could choose which foot was at the front of their stance and they kept the same foot in front in all trials. For all experimental conditions, participants supported themselves with two canes, one held in each hand, their arms comfortably extended and their trunk kept upright (Fig. 1). Participants were asked to apply one of three levels of force on the canes: minimum (Min), i.e., as little as they could, preferred (Pref), i.e., as much as they liked, maximum (Max), i.e., as much as possible. They performed the same three force conditions in two arm configurations: their arms extended out horizontally in the frontal plane (Planar), and stretched out forward forming approximately a 45° angle at the shoulder with the frontal plane (Tripod), midway between the sagittal and the frontal planes. The planar configuration limited the base of support to a line orthogonal to the beam, while the tripod configuration enlarged the base of support to a triangular area. Standing on a narrow beam elicited instability around the vertical, mainly in the frontal plane. Therefore, we chose the planar configuration to counter such effects directly. The rationale for choosing straight arms was twofold: first, this simple joint configuration minimized differences across individuals and, second, it eliminated additional stiffness of the elbow and the wrist joints acting at the hand/cane junction.

In addition, two reference conditions were tested: in the first condition participants stood on the ground in the same tandem stance without holding canes (Off Beam–No Canes). This condition provided a reference to understand the instability induced by the beam. In the second control condition, participants stood on the beam without the cane support (On Beam–No Canes). In this difficult condition they were allowed to move their arms freely to help maintain balance on the beam. Comparisons with the main experimental conditions established a baseline for the mechanical challenges posed by standing on the beam and the supportive effects of the canes when standing on the beam (Hypothesis 0).

Each combination of force on the canes and arm configuration was repeated three times, grouped into two blocks. One block was performed with the planar cane placement, the second one with the tripod configuration. Each block presented 3 trials for each of the 3 force levels (Maximum, Minimum, Preferred). The arm configurations were grouped into two blocks to avoid too much disruption from changing cane placements. Prior to each block, participants performed the two reference conditions, first standing on the ground without canes (Off Beam–No Canes), then standing on the beam without canes (On Beam–No Canes). Each trial lasted 30 s; the entire recording session lasted approximately one hour, including the time to place the markers on the body.

### Data preprocessing

All analyses were carried out with custom software written in Matlab (The Mathworks Inc., Natick, MA, USA). All kinematic and kinetic data were filtered with a zero-lag, 3rd-order, low-pass Butterworth filter at 10 Hz (functions: *butter*, *filtfilt*). In order to exclude any familiarization or fatigue effects, data from the first 10% and last 10% of each trial were excluded from the analysis. The weight of the cane was subtracted from the vertical component of the force measured at the canes to estimate the effective force applied by participants. In the control condition where participants stood on the beam without cane support, they occasionally lost balance and stepped off the beam. These trials were excluded from the analysis.

As the feet of the participant were not in direct contact with the force plate but only with the beam, the center of pressure recorded by the force plate (Ground-CoP) was different from that resulting from the feet-beam interaction (Beam-CoP). The discrepancy was evaluated by the following equations

1 $${\text{Beam-CoP}}_{\mathrm{x}}={\text{Ground-CoP}}_{\mathrm{x}}+h\frac{{F}_{x}^{g}}{{F}_{z}^{g}},$$

2 $${\text{Beam-CoP}}_{y}={\text{Ground-CoP}}_{y}+h\frac{{F}_{y}^{g}}{{F}_{z}^{g}},$$

where *h* is the height of the beam and ${F}^{g}=\left[{F}_{x}^{g},{F}_{y}^{g},{F}_{z}^{g}\right]$ is the ground reaction force recorded by the force plate. The x-axis corresponded to the medio-lateral (ML) direction, the y-axis to the anterior–posterior (AP) direction, and the z-axis to the vertical direction, as illustrated in Fig. 1.

As ${F}_{z}^{g}>> {F}_{x,y}^{g}$, the additional terms on the right side of Eqs. (1) and (2) were negligible. Thus, in the following only the Ground-CoP was considered. For the sake of clarity, the CoP on the ground was referred to as the Feet-CoP.

When two canes touched the floor, the participant had three regions of contact with the ground: the feet on the beam, and the tips of the two canes. The feet were on the beam that was placed on the force platform, thus measuring the ground reaction force and the center of pressure. Information about the force applied on the canes was provided by the load cells at the tip of the canes. The center of pressure of each cane *cop* was computed by the ratio between the moments, $m_{x}$ and $m_{y}$, and the forces, $f_{z}$, measured by the load cells

3 $$cop_{x} = \frac{{m_{y} }}{{f_{z} }},$$

4 $$cop_{y} = \frac{{m_{x} }}{{f_{z} }}.$$

The spatial positions of the tips of the canes were determined from the markers attached to the canes. With all variables in the laboratory coordinate frame, the total center of pressure (Total-CoP) was computed as the ratio of the total moments, $M_{x,y}$, and the total force, $F_{z}$. The total moments were defined as the sum of the product of the vertical force at each point of contact with the respective moment arm. The moment arm at each point was computed as the sum of the center of pressure with the relative position *a* $=\left[{\mathrm{a}}_{\mathrm{x}},{\mathrm{a}}_{\mathrm{y}},0\right]$, which in turn is the vector from the origin of the coordinate frame to the point of contact. As it was desirable to compute the CoP in the medio-lateral (ML) and antero-posterior (AP) directions, the total moments in the AP and ML directions were determined, respectively, as shown in Eqs. (5) and (6)

5 $$M_{x} = \sum\limits_{i = 1}^{3} {(a_{y}^{i} + cop_{y}^{i} )} f_{z}^{i}$$

6 $$M_{y} = \sum\limits_{i = 1}^{3} {(a_{x}^{i} + cop_{x}^{i} )} f_{z}^{i} .$$

The index $i$ indicates the current point of contact (i = 1: feet, i = 2: left cane, i = 3: right cane). Following the rule applied previously, the total CoP was determined as

7 $${\text{Total-CoP}}_{\mathrm{x}}=\frac{{M}_{y}}{{F}_{z}},$$

8 $${\text{Total-CoP}}_{\mathrm{y}}=\frac{{M}_{x}}{{F}_{z}},$$

where $$F_{z} = \sum\limits_{i = 1}^{3} {f_{z}^{i} }$$.

For each participant a kinematic model of 15 rigid body segments (head, trunk, pelvis, left and right upper arms, forearms, hands, thighs, shanks and feet) was fit to the kinematic data using C-Motion Visual3D (Germantown, MD). The whole-body center of mass (CoM) was computed in Visual3D.

### Dependent measures

To obtain a metric for postural sway, the fluctuations of the CoP were summarized by the standard deviations of the CoP in two orthogonal directions, the anterior–posterior (AP) and the medio-lateral (ML) directions. These two directions were calculated separately because of the asymmetric constraints of the beam, i.e., the base of support in the AP direction was significantly larger than in the ML direction. Another measure of postural sway was defined as the area of the 95% tolerance ellipse. The same metrics were computed for both Feet-CoP and Total-CoP. Similarly, the fluctuations of the center of mass (CoM) were quantified by the area of the 95% tolerance ellipse. This area was calculated in the horizontal (x–y) plane to make it comparable to the areas of the CoPs. Note that movements in the vertical (z) direction were negligible. To quantify movements of the hand at the tip of the cane, the path length of the hand movement was calculated as the integral of the root mean squared sum of the derivatives of the x-, y- and z-components,

9 $${\text{path length}}={\int }_{start}^{end}\sqrt{{\left(\frac{dx}{dt}\right)}^{2}+{\left(\frac{dy}{dt}\right)}^{2}+{\left(\frac{dz}{dt}\right)}^{2}}dt .$$

### Statistical analysis

A linear mixed model was used to evaluate the differences in the variability of the CoM and the CoP between the three levels of force (Min, Pref, Max) applied to the canes and the two cane placements (Planar, Tripod). The mixed model compared the experimental conditions (fixed effects), i.e., beam, force, and arm configuration conditions, which were consistent across participants, and accounted for the effects of normally distributed variability between participants (random effects). This linear model allowed the two control conditions (On Beam–No Canes and Off Beam–No Canes) to be included, even though they were not part of the balanced 3 (force levels) × 2 (cane placements) design. To identify the model that best fit each dependent variable, an iterative procedure was adopted to assess whether the inclusion of random effects was justified [18]. Then, according to the hypothesis testing method [19, 20], we iteratively compared different models that assessed whether it was necessary to include interaction terms or random-effect slopes. In Eq. (10), *B* is the beam condition (On Beam–No Canes or Off Beam–No Canes); *F* is the force condition (three levels: Min, Pref, Max), *C* is the arm configuration (two levels: Planar and Tripod), *Y* is the dependent variable for each participant *i* and each trial *j*. $\beta$ are the fixed-effects coefficients, *S* are the random-effects coefficients from the participants,

10 $${Y}_{ij}={\beta }_{0}+{S}_{0i}+{\beta }_{b} {B}_{j} +\left({\beta }_{F}+{S}_{Fi}\right){F}_{j}+{\beta }_{c} {C}_{j} +{\epsilon }_{ij}.$$

To better compare the force conditions in which participants were standing on the beam with the canes on the ground, a second model (see Eq. 11) was tested on a subset of the data, excluding the trials of the two reference conditions,

11 $${Y}_{ij}={\beta }_{0}+{S}_{0i}+\left({\beta }_{F}+{S}_{Fi}\right){F}_{j}+{\beta }_{c} {C}_{j} +{\epsilon }_{ij}.$$

Additional multiple comparisons were conducted across experimental conditions by pairwise t-tests with Bonferroni corrections. The significance level was set to *p* = 0.05.

All statistical analyses were carried out in R, with packages *stats*, *lme4* and *lmerTest* [21].

## Results

### Forces applied on the canes

The first test verified that participants indeed followed instructions and applied different forces on the canes. Table 1 shows the summed vertical forces applied on the two canes averaged over the duration of the trial. For the three force conditions and for the two cane placements the applied forces ranged between 2 and 194 N. The linear mixed model confirmed the difference between the three force levels with a significant main effect (*β* = 10.1 ± 2.6, *p* < 0.001). All three force conditions were larger than those examined in previous studies and the preferred force differed from both the maximum and the minimum forces. The two arm configurations did not elicit different forces in the three force conditions (*β* = − 0.7 ± 4.3, *p* = 0.86).

**Table 1:** Sum of forces applied on the two canes

Force	Min (M ± SD)	Pref (M ± SD)	Max (M ± SD)
Arm Configurations			
Planar	8.25 ± 10.08 N	33.21 ± 11.97 N	91.20 ± 36.82 N
Tripod	9.64 ± 12.15 N	32.87 ± 12.28 N	85.60 ± 31.71 N

Means and standard deviations across participants of the sum of the forces applied on the two canes in the three force conditions and in the two cane placements. Forces were averaged across the duration of the trial and then summed for the two canes

### Center of pressure and center of mass in control and experimental conditions

**Fig. 2:**
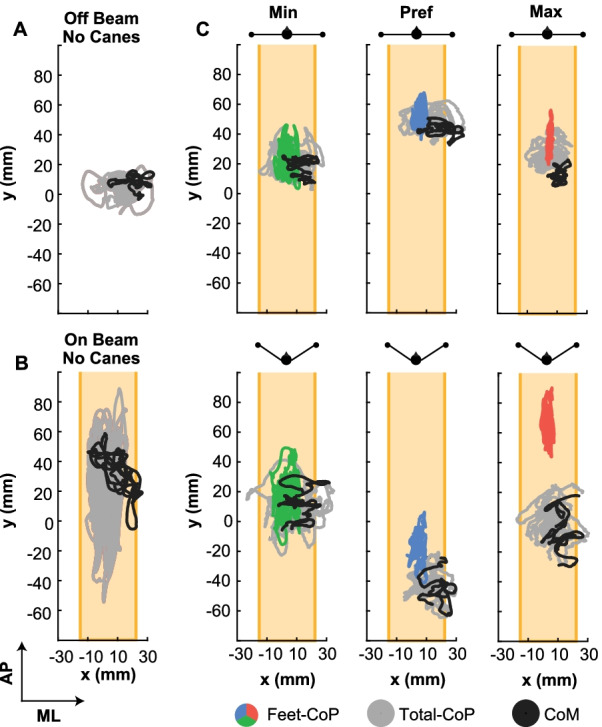
Representative paths of the center of pressure (CoP) and of the center of mass (CoM) in the horizontal x–y-plane. The Feet-CoP, Total-CoP and the CoM of one trial for each of the three force instructions and the two arm configurations are shown. **A** Exemplary trial when standing on the ground in tandem stance (Off Beam – No Canes). The grey line represents the CoP and the black line the CoM. **B** CoP and CoM of one trial of the same participant when standing on the beam without canes (On Beam – No Canes). **C.** Six panels showing both the Feet-CoP (colored) and the Total-CoP (grey) for the three force conditions: minimum (green), preferred (blue), maximum (red); black lines show the center of mass (CoM). The two cane configurations are identified by the drawings at the top of each panel. The beam is the light yellow area bounded by thin lines for visibility. For all conditions on and off the beam, the participant stood in tandem stance with the same foot in the front

Figure 2 displays exemplary trials of Feet-CoP (colored lines), Total-CoP (grey lines) and also of the CoM (black lines) for each experimental condition (yellow shading represents the beam width). Compared to standing on the ground (Off Beam–No Canes, Fig. 2A), the fluctuations of CoM and CoP were considerably higher when standing on the beam without canes (On Beam–No Canes, Fig. 2B). In the latter condition, both CoP and CoM showed visibly larger excursions, both in the AP and ML directions, providing evidence that the beam induced considerable instability.

The six panels in Fig. 2C show exemplary data from the same participant standing on the beam but now with the canes on the ground. The excursions of both CoPs and CoM were significantly reduced compared to those when standing on the beam without cane support (Hypothesis 0). More interestingly, they became similar to those fluctuations measured when standing on the ground (Fig. 2A). Further, in the minimum force condition, variability in the x-direction (ML) was similar in both Feet-CoP and Total-CoP, in both cane placements. With increasing forces applied on the canes, the Feet-CoP decreased its ML amplitude. In contrast, the Total-CoP went beyond the width of the beam, indicating that the participants were moving their weight away from the feet and were actively relying on the canes. The tripod configuration led to visibly higher variability in the y-direction (AP) than the planar configuration, especially when applying maximum force onto the canes. Lastly, the fluctuations of the CoM, shown by the black lines, followed the changes of the Total-CoP across different forces and cane placements and presented additional evidence that participants shifted their weight beyond the base of support on the beam towards that provided by the canes.

It can also be observed that the CoP location along the beam changed between trials, even within the same participant. This effect resulted from changing the distribution of body weight between the front and the back foot, even without stepping off the beam between trials.

### Comparison of postural sway on and off the beam

**Fig. 3:**
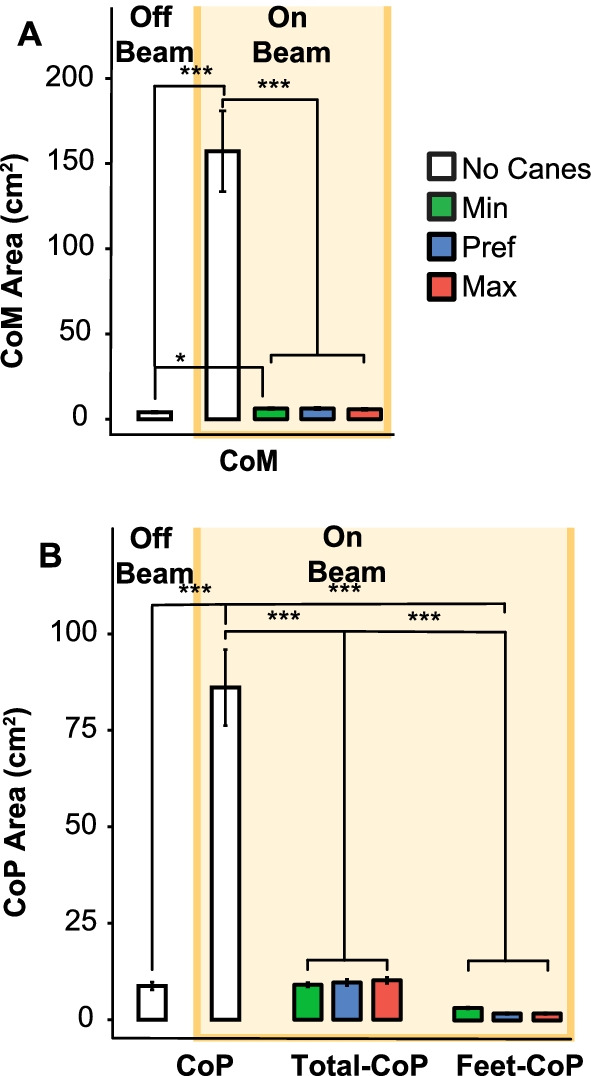
Postural sway quantified by the area of the 95% tolerance ellipse for the center of pressure (CoP) and center of mass (CoM) for all experimental conditions. The white background on the left shows results for standing on the ground, the light yellow background indicates metrics for standing on the beam. Each bar shows the mean and standard error (n = 16) for the different experimental conditions, pooled over all participants. The colored bars show the metrics when the participants used canes; green, blue and red differentiate the three force conditions. **A** Area of the center of mass (CoM) quantified by the 95% tolerance ellipse. The white bars show the CoM area when participants stood on the ground and on the beam, without canes; the green, blue and red bars represent the three force conditions. **B** Area of the center of pressure (CoP, Total-CoP and Feet-CoP) quantified by the 95% tolerance ellipse. The two white bars show the CoP area when participants did not use canes. The lower value of CoP on the left represents the participants standing on the ground; the white bar shows the CoP area when participants stood on the beam. The colored bars show the Total-CoP and the Feet-CoP when the participants used canes. (significance levels: ***p < 0.001; *p < 0.05)

To first evaluate the difference between balancing on the beam supported by canes with the two control conditions, the 95% tolerance ellipse of the CoM served as a collective measure of balance performance. Figure 3A shows the CoM in the two control conditions on and off the beam (white) next to the experimental conditions on the beam with three force levels (colored); the data combined the two arm configurations to focus on the comparison with the two control conditions. The figure makes it evident that standing on the beam without canes had the highest degree of variability as to be expected (*β* = 1682.1 ± 290.2, *p* < 0.001). More notable is that when standing on the beam with canes, the variability of the CoM decreased to levels similar to the variability on the ground. Additionally, when comparing to the different force conditions with pairwise post-hoc comparisons, the two higher forces did not differ from standing on the ground (Pref: *p* = 0.08, Max: *p* = 0.1). Only the minimum force condition showed a small but significant elevation compared to standing on the ground (Min: *p* = 0.02). When applying increasing force on the canes, the variability of the CoM did not change (p = 1).

Figure 3B shows participant averages of the 95% tolerance ellipse of the CoP for the two control conditions without cane support (white bars) next to the three force conditions (colored). To take into account the different nature of Total-CoP and Feet-CoP when participants used canes, the results were separated. Again, the data were pooled for the two arm configurations to facilitate comparison. As expected, standing on the beam without cane support significantly increased the CoP excursions with respect to standing on the ground by a factor of 10 (β = 2953.6 ± 236.2, *p* < 0.001). However, when participants used the canes for support, the Total-CoP returned to values similar to standing on the ground, as confirmed by the pairwise post-hoc comparisons (p = 1). The Feet-CoP showed even smaller values than the Total-CoP with canes and the CoP on the ground without canes (p < 0.001).

Taken together, these findings quantified and confirmed expectations that standing on the beam significantly increased CoP and CoM motions and that canes reduced postural instability (Hypothesis 0). With the support of canes both CoM and CoP variabilities returned to those when standing still on the ground without canes. Given the high variability when balancing on the beam without canes, this gives first evidence of the significant mechanical effect of the canes.

### Effect of forces on postural balance in the ML direction

**Fig. 4:**
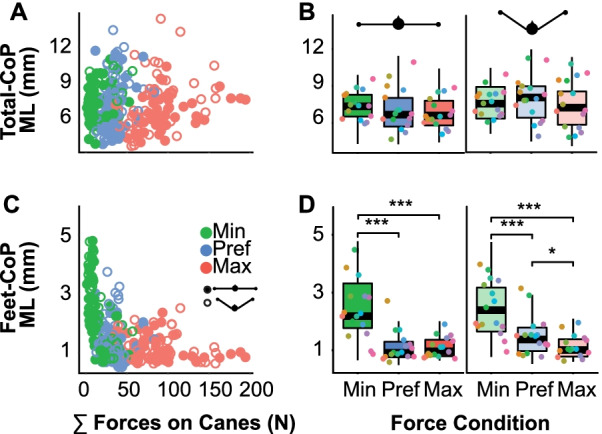
Total center of pressure over feet and canes (Total-CoP) and center of pressure at the feet (Feet-CoP) in the medio-lateral (ML) direction for the three force conditions. **A** Medio-lateral (ML) component of the Total-CoP motion with respect to the sum of the forces applied on the canes; each data point is the average of one trial. Filled circles represent the planar configuration, empty circles the tripod configuration. **B** Standard deviations of the ML component of the Total-CoP motion for the two arm configurations. Each data point represents one participant; different colors indicate different participants. **C** Feet-CoP in the ML direction against the sum of the forces applied on the canes for each trial. **D** ML component of the Feet-CoP for each force condition and for the two arm configurations. Each data point represents one participant, different colors indicate different participants. (significance levels: ***p < 0.001; *p < 0.05)

Having related the findings with respect to the control conditions, the next analyses focused on the experimental conditions when the participants stood on the beam and exerted three different force levels onto both canes. The main metric is variability in the ML direction, computed as the standard deviation of the CoP motion, as it is the most relevant direction for maintaining balance on a beam. Figure 4A shows the ML variability of the Total-CoP against the average forces applied on the canes; the data points represent all individual trials of all participants with force condition differentiated by color. Figure 4B shows the same data averaged across the different force levels and cane placements and pooled over all participants. There was no evidence of any change with increasing force (β = − 0.000015 ± 0.0001, *p* = 0.87). While different from what was expected in Hypothesis 1, this finding was consistent with previous results: ML variability was significantly attenuated with small forces at the support, and increasing force levels did not further affect the Total-CoP [3, 7]. However, the Total-CoP was affected by the cane placement showing a slightly larger ML variability in the tripod condition (β = 0.0006 ± 0.0002, *p* < 0.001). This effect of arm configuration was counter to Hypothesis 2.

In contrast, the ML standard deviation of the Feet-CoP decreased with the average force for each trial, as shown in Fig. 4C. Figure 4D shows the pooled data of all participants for each experimental condition. For both cane placements, the same trend was observed: applying a force larger than minimum force reduced Feet-CoP variability (β = − 0.0008 ± 0.00015, *p* < 0.001). This indicates that increasing force applied on the canes let participants rely less on the foot-beam interaction and more on the canes. The variability in the ML direction for the Feet-CoP was not affected by cane placement (β = − 0.00016 ± 0.0002, *p* = 0.45), indicating that the spatial configuration of the arms was not relevant for the ML direction of the Feet-CoP. This set of results on Feet-CoP was consistent with Hypothesis 1 and 2.

### Effect of forces on postural balance in the AP direction

**Fig. 5:**
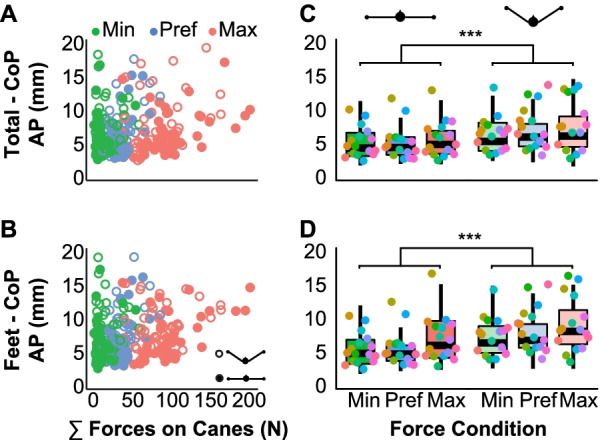
Total center of pressure (Total-CoP) and center of pressure at the feet (Feet-CoP) in the anterior–posterior (AP) direction for different cane conditions. **A** Anterior–posterior (AP) component of the Total-CoP motion with respect to the sum of the forces applied on the canes; each data point is the average of one trial. Filled circles represent the planar configuration, empty circles the tripod configuration. **B** Feet-CoP in the AP direction against the sum of the forces applied on the canes for each trial. **C** Standard deviations of the AP component of the Total-CoP motion for the two cane configurations. Data are pooled together for each force condition. Each data point represents one participant; different colors indicate different participants. **D** AP component of the standard deviation of the Feet-CoP for each force condition and for the two cane conditions. Each data point represents one participant, different colors indicate different participants. (*** indicates significance of *p* < 0.001)

The standard deviations of both Feet-CoP and Total-CoP were also compared in the AP direction, i.e., along the beam length. Figures 5A, B show respectively the Total-CoP and the Feet-CoP in the AP direction for each individual trial. Applying different levels of force did not affect AP variability neither in Feet-CoP (β = 0.0003 ± 0.0002, *p* = 0.29) nor in Total-CoP (β = 0.0002 ± 0.0002, *p* = 0.37) as indicated in the boxplots of Fig. 5C, D. However, AP variability was larger in the tripod condition than the planar condition, both for Feet-CoP and Total-CoP (Feet-CoP: *β* = 0.002 ± 0.0007, *p* < 0.01; Total-CoP: *β* = 0.002 ± 0.0004, *p* < 0.001). This confirmed that the tripod cane placement allowed for more variability along the length of the beam and that participants actually exploited this extended base of support (Hypothesis 2).

### Effect of forces on variability of cane motion

**Fig. 6:**
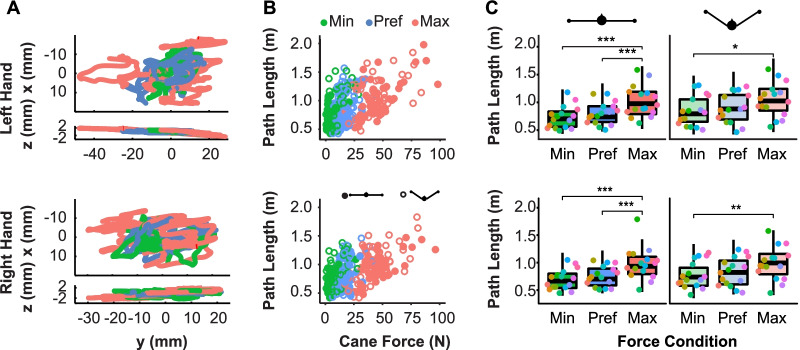
Paths and path lengths of the left and right hands for different force instructions differentiated by color. **A** Exemplary paths of the movements of the left and right hands from two points of view: x–y at the top, z–y below. Each colored line shows one trial in the three force conditions. **B** Path lengths for the left and right hands per trial plotted against the average force applied to the cane. Each point indicates an individual trial. **C** Path length for both hands aggregated with respect to force levels and cane configurations. Each data point represents one participant, different colors indicate different participants (*** indicates significance of *p* < 0.001)

Even though participants were instructed to stand still, some small movements of the body, arms, and hands were always present [22]. Noise coupled to the compression force inevitably transferred from the hand to the cane handle, deflecting the cane from the vertical position. To quantify these deflections, the path length of the hand on the cane handle was computed for each trial. Figure 6A shows the paths traveled by the right and left hands of one participant over the course of one trial in each force condition (marked by color). The path length of the hand increased when more force was applied (right cane: *β* = 0.006 ± 0.001, *p* < 0.001; left cane: *β* = 0.006 ± 0.001, *p* < 0.001). Figure 6B shows path lengths of all participants, plotted against the average force applied on the respective cane; each point represents the path length traveled by the right or left hand during one trial. It shows that the path length increased with the amount of force applied. Path length in the minimum force condition was significantly different from the maximum force condition for both hands (right hand: *p* < 0.001, left hand: *p* < 0.001). The preferred force condition was not significantly different from the minimum condition for the right hand (p = 0.09) and only slightly significant for the left hand (*p* < 0.05), as indicated in Fig. 6C. Nonetheless, arm configuration also played a role in cane stabilization as path length was slightly larger in the tripod configuration for both hands (right cane: *β* = 0.0006 ± 0.0001, *p* < 0.001; left cane: *β* = 0.0008 ± 0.0001, *p* < 0.001). These results suggest that higher forces indeed had destabilizing side-effects as expected (Hypothesis 3).

## Discussion

Complementing much previous work on the role of light touch in postural balance, this study detailed the mechanical effects of the canes on postural control in a challenging balance task. Specifically, the experiment aimed to identify the mechanical effects of two canes on the control of balance when standing on a narrow beam. Participants were asked to exert three levels of force onto the canes while placing two canes either to the sides or in a triangular configuration with the feet. The overarching question of this study was how the different forces applied to the canes and two arm configurations affected the mechanics and, hence, the control of postural balance.

Overall, while standing on a narrow beam, the use of canes improved postural balance as evidenced by the reduced variability of the CoP and the CoM (Hypothesis 0). These fluctuations declined to the same level as when standing on the floor, demonstrating that canes had a beneficial effect on postural balance. This reduction of postural sway was observed for all force levels. While applying more force on the canes did not affect the medio-lateral (ML) component of the Total-CoP, it did affect the Feet-CoP that was measured directly under the feet on the beam (Hypothesis 1). Having the canes in triangular arrangement increased variability in the antero-posterior direction, while the medio-lateral direction remained relatively unaffected by this cane placement (Hypothesis 2). However, when exerting higher forces signs of destabilization emerged, as the inverted pendulum nature of the canes was susceptible to small excursions at the handle (Hypothesis 3).

### Perceptual benefits of canes

Numerous previous studies investigated the effect of light touch on postural control and showed that even when increasing forces applied on the support surface the CoP motion was not further reduced [3, 7, 12, 23–25]. However, the touch conditions in these previous studies by Jeka and colleagues remained intentionally low, in the range between 1 and 5 N. Naturally, as the authors intended to focus on perceptual information, they avoided confounding with other mechanical effects. Further, they instructed participants to apply this small force to match a target level. This might have created additional control processes beyond maintaining balance. To avoid this possibility, this study did not provide participants with feedback of their applied force, but left them free to choose the amount of force, as long as they chose three different levels. Our results on variability of the Total-CoP and the CoM showed again that, while canes were generally helpful for balance, exerting the maximum level of force on the canes did not provide any further stabilizing effect. These findings corroborated the widely accepted conclusion that touch, even for very low contact forces, provides perceptual information that enhances balance performance, similar to how visual information stabilizes postural control [1]. Hence, at first blush, these results seem to contradict the Hypothesis 1 that the mechanical effects of the support benefitted postural stability.

Note that it is not straightforward to extricate perceptual from mechanical effects as these two aspects are always present and are tightly intertwined. One can neither eliminate perceptual contributions in human subjects, nor eliminate basic mechanics. However, the typical experimental method is to exaggerate one contribution over the other. Therefore, our study manipulated the instructed forces exerted on the canes to go beyond the forces that have been previously measured in the context of perceptual support. The cane placement was a second way to change the mechanics of the posture. These two factors in the experimental design explicitly manipulated aspects that were expected to elevate the role of mechanics in the task. It is worth noting that there was no noticeable adaptation throughout the experiment, as the statistical analysis did not identify any significant effect of trial number (p > 0.05, see footnote^1^). This absence of any improvement further underscores that the differences in CoP with increasing force levels and arm configurations were mainly induced by changes in mechanics. This lack of change in behavior throughout the experimental session is consistent with what was previously observed in balancing on a beam wearing rigid soles [26].

### Mechanical benefits of canes for balance control

Intuitively, canes should facilitate balance as canes on the ground increase the base of support, which inherently changes the mechanics of the system. But what are these mechanical effects and how do they affect demands on postural control? Our data gave several indications that cane support went beyond being purely perceptual support and also afforded mechanical benefits. First, as the additional contacts with the floor enlarged the base of support, CoP and CoM went outside the beam width. Therefore, humans indeed used the available larger base of support.

Second, the Feet-CoP motion significantly decreased with increasing force on the canes, indicating that the more force applied on the canes, the more control relied on their support to balance.

Third, while the extent of the fluctuations in CoP and CoM did not depend on the magnitude of forces applied to the canes, they did depend on the placement of the canes. The standard deviations of the Total-CoP and Feet-CoP in the tripod condition were significantly larger than in the planar configuration. In particular, the tripod placement affected predominantly the anterior–posterior (AP) direction.

Fourth, the preferred force applied on both canes was reliably around 33 N in both arm configurations, corresponding to a weight of a 3.36 kg mass. Assuming the weight of one arm is 5% of their total body weight, this force approximated the weight of the arm for a body mass of 67 kg (3.35 kg) [27, 28]. To probe into this effect, we estimated the weight of each participant’s arm as 5% of their total body mass and correlated them with the sum of the forces applied on both canes (calculated as the average across time and divided by gravity). We further assumed that the weight of each arm would be supported by both the shoulder and the cane. Therefore we considered half of the arm weight for each arm (see Eq. 12),

12 $$\frac{1}{2}{mass}_{left arm}+\frac{1}{2}{mass}_{right arm}\propto {F}_{left cane}+{F}_{right cane.}$$

**Fig. 7:**
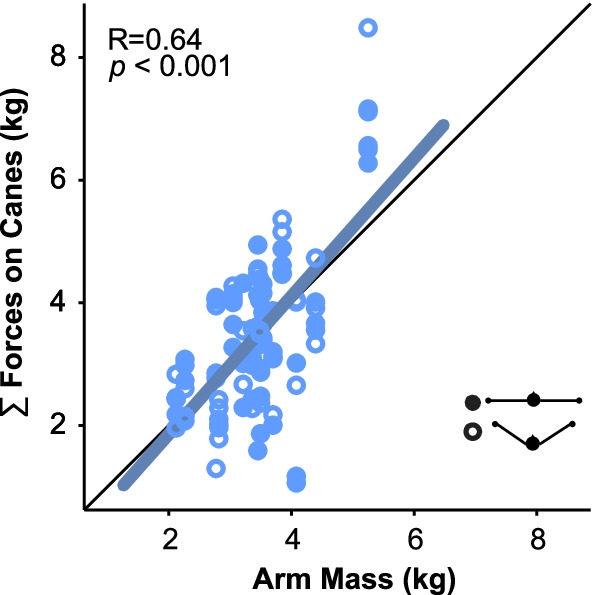
Relation between estimated arm mass and force applied on the canes in the preferred force condition. Each data point represents the value for one trial (3 trials per participant). Arm mass was estimated as 5% of the total body mass of each participant. The force values were computed as the average force applied on one cane during one trial plus the average force applied on the other cane. This force value was divided by the gravitational acceleration (9.81 m/s^2^) to be compared with the corresponding arm mass. Filled and empty points are trials in the planar and tripod configurations, respectively. The Pearson correlation coefficient indicates a significant correlation shown by the blue line; the black line is the identity line

The data in Fig. 7 cluster very closely around the identity line, indicating that the preferred force was determined by the weight of the participants arms (r = 0.64, *p* < 0.001). Hence, this preferred force offset the need to hold the arms against gravity and thereby reduced the effort required to hold one’s arms, exploiting the new mechanical support provided by the additional devices.

### Mechanical challenge due to instability of the canes

Applying forces on the vertical canes is an isometric task with potential instability. Unlike in previous studies that tested forces applied on a fixed surface, the canes were not inherently stable; rather, mechanically they presented an inverted pendulum at its unstable equilibrium point. Hence, the inherent noise in the human sensorimotor system introduces displacements. Assuming that noise increases with force, this destabilizing effect increases with higher forces [15]. On the other hand, as previously shown in the context of pushing a stick against a wall, a downward compression force component on the cane increases the instability of the inverted pendulum and any small excursion will destabilize the canes [16, 17, 29]. Human joint stiffness also increases with increasing force and may have counteracted this perturbing effect to maintain postural balance [30]. However, given the straight arms in our experiment, the stiffness at the shoulder joint may have been limited as the increased displacements of the hands and canes with higher forces showed. Interestingly, the variability of the CoM and the Total-CoP did not vary with increasing force, indicating that the larger displacements at the hand may have been compensated at the torso. Hence, these findings revealed that the cane support not only facilitated balance, but also created complex control demands across the multi-segmented body.

### Underlying control mechanisms

All together, these results present an intricate picture of how the canes affected the control of postural stability, with some effects potentially cancelling each other. Note that we use the term stability in the sense of remaining in the vicinity of a nominal standing posture despite unavoidable fluctuations. What are the control mechanisms underlying these observations? To begin, when standing on the beam with canes, the variability of the CoM and Total-CoP in the task-relevant ML direction were essentially identical to those when standing on the ground. If the measured fluctuations when standing on the ground are regarded as a floor effect determined by the noise level (as participants were asked to stand as still as possible), then the use of canes enabled participants to minimize CoM and Total-CoP variability to this lower bound. That could also be the reason why the different force levels did not lead to further reductions.

Second, when the canes formed a tripod, the fluctuations of the CoP were larger than in the planar condition, making use of the larger base of support. This observation is consistent with the notion that in a task with redundancy, variability is channelled into a task-irrelevant dimension [31, 32]. The numerous degrees of freedom of this whole-body task can be configured into infinitely many ways to keep the CoM and CoP over the base of support. While motion of the Feet-CoP in the ML direction is clearly bounded by the edge of the beam, higher variability of the CoP in the AP direction does not jeopardize the participant’s posture on the beam. This suggests that the controller did not constrain fluctuations, i.e., allowing more variability in this task-irrelevant direction. Allowing variability in directions orthogonal to what affects the task is usually interpreted as a reduction of control effort [33]. In sum, the controller allows fluctuations as long as the CoP stays within the limits defined by the margin of the base of support and by the noise of the system.

Third, control took advantage of the additional devices, evidenced in the preferred force level that just off-set the weight of the arms while staying away from higher forces that potentially introduced destabilizing effects. We speculate that the controller avoids higher forces not only because they require more effort without any obvious benefit, but also because they introduce additional demands on neuro-muscular stiffness to counteract destabilizing forces.

### Limitations, implications and outlook

In the present study participants used canes to balance on a narrow beam holding them with the arms extended. While this presented a clean geometric body configuration, different mechanisms might be manifest if the canes were held with flexed arms as may be more convenient in real life. Our metrics, ML and AP standard deviations and the total area of CoM and CoP, could capture interesting features of the task, but they were scalar measures of data distributions. Additional analyses could characterize the temporal evolution of the forces and their relative centers of pressure. Recent work went beyond analyzing the point of application of the force vector, and examined the orientation of the ground reaction force with respect to the center of mass. This analysis revealed interesting information about the relative role of biomechanics and control [34, 35]. Applying these methods to the more challenging task of standing on a narrow beam with canes could provide further information about the strategy adopted by the controller when using canes.

Our study afforded a clean analysis highlighting the intricate mechanical effects that a user can exploit and has to compensate for. Awareness of these effects is important for the design of canes and the instruction of how to use them. Note that while walkers are more stable, when moving in small spaces and on uneven ground, frail people will still need to support themselves with canes. Understanding the mechanical together with the perceptual effects of canes is therefore important for elder care and rehabilitation.

## Conclusions

Postural balance improves with light touch on a stable surface suggesting perceptual benefits of additional support. Here, new insights on the mechanical benefits of inherently unstable devices—canes—are presented. Participants adapted to the novel mechanical system by trading off the benefits from the additional support with the instability introduced by pushing on the canes. Fluctuations of ground reaction forces show a channeling of noise in the task-irrelevant dimensions. Such mechanical benefits provide a better understanding of the role of support when balance is challenged, allowing future work to explore tailored rehabilitation protocols and the development of novel assistive devices.

## Data Availability

The datasets used and/or analysed during the current study are available from the corresponding author on reasonable request.

## Abbreviations

CoP: Center of pressure
CoM: Center of mass
AP: Antero-posterior
ML: Medio-lateral

## Footnotes

1 As the sequence of conditions was not randomized, we therefore examined whether there was any familiarization or learning effect across the experimental trials. The CoP variables of all individual trials for all different conditions were examined for trends. The linear mixed model could not identify any improvements: Feet-CoP ML: *β* = − 0.00009, *p* = 0.993; Feet-CoP AP: β** = **0.085, *p* = 0.170; Total-CoP ML: *β* = − 0.01, *p* = 0.555; Total-CoP AP: *β* = 0.090, *p* = 0.118. Note that this absence of any improvement is understandable as maintaining balance is an ability that may have reached a plateau as it is inherent to most everyday activities. Any improvements beyond the daily needed level are likely to require long hours of practice as seen in tight rope artists and gymnasts.
